# Energy harvesting from enzymatic biowaste reaction through polyelectrolyte functionalized 2D nanofluidic channels†
†Electronic supplementary information (ESI) available: Experimental section, electrical measurement setup and composite membrane characteristics. See DOI: 10.1039/c5sc04634c


**DOI:** 10.1039/c5sc04634c

**Published:** 2016-02-16

**Authors:** Lei Lin, Ling Zhang, Lida Wang, Jinghong Li

**Affiliations:** a Department of Chemistry , Beijing Key Laboratory for Analytical Methods and Instrumentation , Tsinghua University , Beijing 100084 , China . Email: jhli@mail.tsinghua.edu.cn

## Abstract

Effective recycling of biowaste energy in a compact system remains a great challenge. Here, we report a graphene-based energy harvesting system powered by enzymatic biowaste reaction through two-dimensional (2D) nanofluidic channels. The integrated 2D nanofluidic generator shows distinct advantages such as flexibility, low cost, and high output in ionic currents.

## Abstract 2

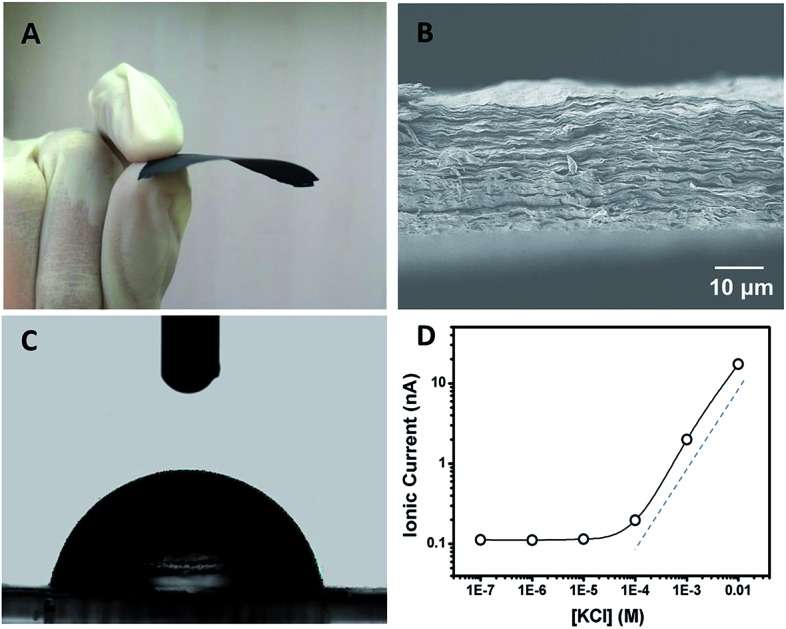
We report a graphene-based energy harvesting system powered by enzymatic biowaste reaction through two-dimensional (2D) nanofluidic channels; the integrated 2D nanofluidic generator shows distinct advantages such as flexibility, low cost, and high output in ionic currents.

## 

There is widespread concern on providing power to the global economy in an efficient and clean manner.^1^ A way of addressing the sensitive issue may lie in the development of biowaste as an alternative energy resource. The exploration of biowaste materials as a renewable energy resource in a compact and integrated system still remains a great challenge. In recent years, two-dimensional (2D) layered materials have been considered as innovative candidates for the construction of energy conversion systems.^2^ Owing to their 2D layered structure, superior mechanical and electrochemical performance,^3^ they exhibit surface-charge-governed ion transportation as the basis for power generation. Functionalized graphene sheets have high flexibility and can be horizontally extended to tens of micrometers.^4^ These unique properties make restacked graphene paper promising in the construction of nanofluidic channels and nanoporous materials for membrane-based technologies, such as microbial fuel cell (MFC) and reverse dialysis (RED).^5^ Compared to conventional porous materials consisting of 1D or 3D fluidic channels, functionalized 2D layered materials provide much more straightforward ways to regulate the inner configuration of the fluidic channels and, therefore, ion transportation property.^6^ Guo *et al.* reported a nanofluidic energy conversion system based on electrokinetic ion transport through a layered chemical converted graphene (CCG) membrane.^7^ They were able to convert mechanical energy in the water flow into electricity with the membrane-based device. However, to date, the recycling of biowaste energy through 2D nanofluidic systems remains unexploited.

Here we demonstrate a graphene-based nanofluidic energy harvesting system powered by an enzymatic biowaste reaction (Scheme 1). Polyacrylic acid (PAA) functionalized graphene composite membrane consisting of numerous interconnected 2D nanochannels between graphene layers, forms a negatively charged 2D nanofluidic network within the membrane, that endows the membrane material with cation-selectivity. Negatively charged graphene–PAA composite membranes (GPM) preferentially permeate cations while excluding anions. When urea molecules are catalyzed by urease, they release anions (OH^–^, HCO_3_^–^) and cations (NH_4_^+^). The NH_4_^+^ ions migrate across the membrane under the force of chemical gradients from the reaction chamber to the receive chamber, resulting in a net ionic current (*I*_c_) and charge imbalance across the membrane (measured as a transmembrane electrical potential difference, *E*_m_). When the ionic flow goes through the membrane in vertical direction driven by the enzymatic biowaste reaction, energy conversion is generated as a net transmembrane ionic current. Additionally, this design strategy of the graphene-based 2D nanofluidic system can be generally extended to other 2D and polyelectrolyte materials for smart nanofluidic electrogenic devices and clean energy.

**Scheme 1:**
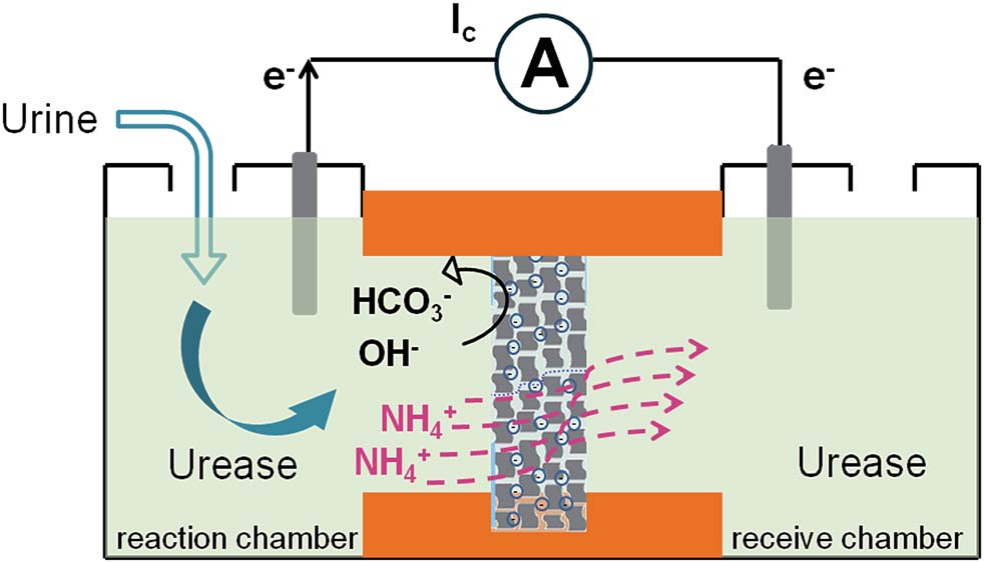
Schematic representation of the biowaste-powered graphene nanofluidic generator. Negatively charged graphene–PAA composite membranes (GPM) preferentially permeate cations while excluding anions. When urea molecules are catalyzed by urease, they release anions (OH^–^, HCO_3_^–^) and cations (NH_4_^+^). The NH_4_^+^ migrate across the membrane under the chemical gradients from the reaction chamber to the receive chamber, resulting in a net ionic current (*I*_c_) and charge imbalance across the membrane (measured as a transmembrane electrical potential difference, *E*_m_).

Polyacrylic acid functionalized graphene composite membrane (GPM) was prepared by vacuum filtration of dispersed graphene–PAA complex colloid solution through a poly(vinylidene fluoride) (PVDF) filter film^8^ (ESI†). The dispersion of GO and PAA was reduced by hydrazine and then filtered through a PVDF filter membrane by vacuum filtration. The as-prepared graphene–PAA composite membrane was peeled off from the filtration film. As can be seen from Fig. 1A, the resultant GPMs are self-supporting. The thickness of the GPM can be readily tuned between 15 and 50 μm by applying appropriate amounts of graphene–PAA dispersion for filtration. The SEM image in Fig. S1B† shows the surface topography of GPM. It is observed that the graphene film exhibits uniform morphology over large areas with wrinkles on the surface.^9^ The cross-sectional view (Fig. 1B) exhibits a uniform layered structure of the freeze-dried GPM. The thickness of the membrane is about 25 μm from the cross-sectional view of SEM in Fig. 1B. Since the XRD pattern of the GPM does not show a prominent peak, the interlayer spacing of the GHM can not be calculated from the characteristic XRD pattern. Additionally, the GPM is macroscopically hydrophobic (CA = 82.4°) and is not dissolved in water (Fig. 1C). Electrostatic repulsion and steric hindrance from negative-charged polymers act as effective spacer factors to counter-balance the interlayer van der Waals interaction, which prevents the restacking of graphene sheets. We have employed attenuated total reflection Fourier transformed infrared (ATR-FTIR) spectra to investigate the surface modification of the graphene sheets. It is observed from Fig. S2† that both PAA and graphene–PAA (G–PAA) show two characteristic peaks at 2928 and 2867 cm^–1^, assigned to the C–H stretching frequencies of PAA. In comparison, GO shows very weak absorption bands at the same region. The obvious signal difference indicates the successful functionalization of PAA onto the surface of graphene. Futhermore, it is known that chemically reduced graphene obtained through the hydrazine method precipitates as agglomerates owing to their hydrophobic nature. Our study shows that, with the assistance of negatively charged polymer, the graphene–PAA mixture is well-dispersed even after chemical reduction (Fig. S1A in ESI†). The above-mentioned results indicate the successful functionalization of PAA onto the surface of graphene, which is also consistent with previous reports.^10^

**Fig. 1:**
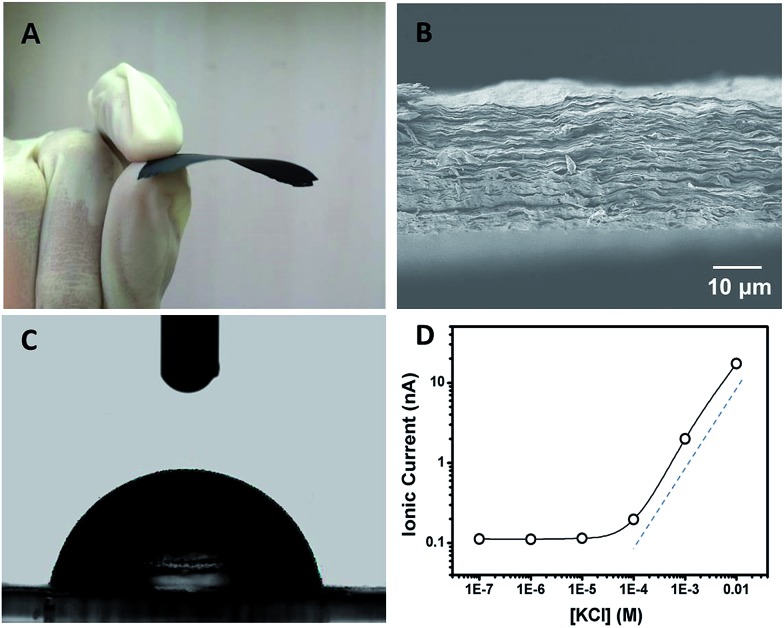
(A) A photograph of the self-supporting and flexible GPM after peeling off from the filtration film. (B) SEM image of the cross-section of a freeze-dried GPM showing layered microstructure. (C) Contact angle (CA) measurement of the GPM with a static CA of *ca*. 82.4°. (D) Concentration-dependent ionic conductance showing surface-governed ion transport at lower salt concentration.

In order to investigate the ion transport properties through the GPM, the composite film was mounted between two chambers of the testing cell filled with electrolyte solution. The transmembrane ionic conductance gradually decreased with the electrolyte concentration from 0.01 to 10^–4^ M, and then reached equilibrium below 10^–4^ M (Fig. 1D), suggesting a surface-charge-governed ionic transport behavior at lower salt concentration.^11^

Fig. 2 shows the typical response of the energy conversion under enzymatic biowaste reaction. Ag/AgCl electrodes were placed in a buffer solution (0.01 mM KCl, 0.5 mg mL^–1^ urease, pH 7), which was connected with the test chamber through an agar-saturated KCl salt bridge (Scheme S1†). This setup ensured the stability of the Ag/AgCl electrode, so that the measured ionic current (*I*_c_) is solely generated from the enzyme-triggered ion transport through the graphene nanochannels. The transmembrane potential (*E*_m_) and the diffusion current (*I*_c_) were recorded as a function of time elapsed (Fig. 2). Without enzymatic catalysis, *E*_m_ and *I*_c_ tend to be constant. After the addition of urea, the catalytic reaction results in a significant rise in both *I*_c_ and *E*_m_. This phenomenon may be attributed to two main reasons. First, the assembled and rich-wrinkled structure of graphene forms numerous 2D nanochannels between neighbouring graphene sheets. All these nanochannels interconnect with each other and finally construct a 2D nanofluidic network within the membrane. Second, the modification with PAA introduces a large number of –COOH groups onto the graphene surface. Under neutral conditions, negatively charged PAA polymer effectively regulates the surface-charge-governed ion transportation of the 2D nanochannels. When the urea molecules are catalyzed by urease, numerous anions OH^–^, HCO_3_^–^ and cations NH_4_^+^ are released instantaneously. Forced by the concentration gradients, the produced NH_4_^+^ transports across the membrane from the reaction chamber to the receiving chamber, resulting in net ionic currents (*I*_c_) and charge imbalance on both sides of the film (*E*_m_). Peak values of 851 nA and 9.72 mV can be reached within 8 min, suggesting the complete degradation of urea, and the electric power density reaches 2.7 mW m^–2^. Based on our experimental data and a previous report,^12^ the maximum voltage (absolute value) and energy conversion efficiency (*η*_e_) were calculated as 177 mV and 0.3%, respectively (Scheme S2†). These results indicate that the as-proposed graphene nanofluidic generators convert the energy of enzymatic biowaste reaction into electricity.

**Fig. 2:**
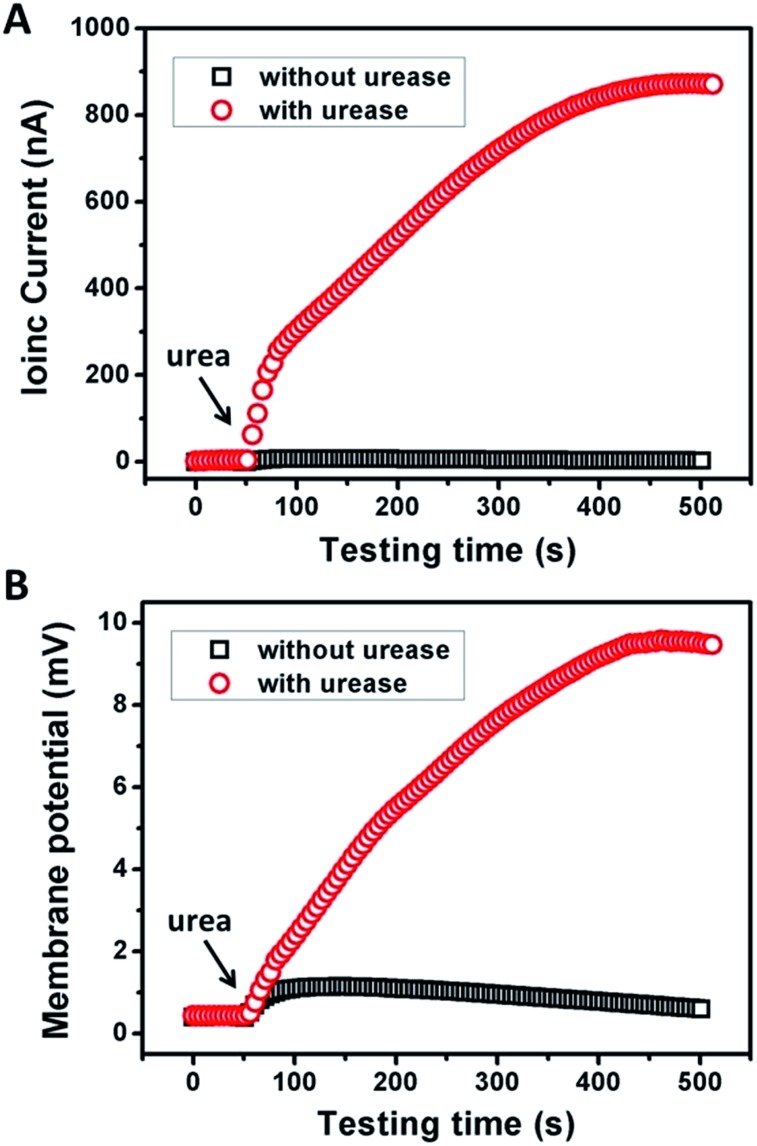
Response of (A) ionic current and (B) membrane potential of GPM under urea triggered enzymatic catalysis. Electrolyte: 0.01 mM KCl, pH 7. The concentrations of urease and urea were 0.5 mg mL^–1^ and 10 mM, respectively.

From the above discussion, we can see that the electrical response correlates with two main factors: the pH and concentration of the electrolyte solution. Fig. 3A demonstrates the current and membrane potential characteristics of GPM in 0.01 mM KCl solution buffered from pH 5 to 9. It is observed that the maximum signal appears at pH 7. As mentioned above, the inner surface of GPM is rich in carboxyl groups. Thus it is highly negatively charged under neutral conditions since the p*K*_a_ of PAA is estimated to be 4.7.^13^ In other words, the carboxyl groups assembled on the graphene surface endow the 2D nanochannels with remarkable cation-selectivity.^14^ Considering the dependence of negative charge density on pH, the 2D nanochannels permeate NH_4_^+^ much more rapidly under higher pH conditions,^15^ leading to higher ion current. On the contrary, excessively high pH cannot produce larger current and membrane potential generation. The reason is that the optimum pH of urease is 7.4 and its activity gradually declines along with increasing pH.

**Fig. 3:**
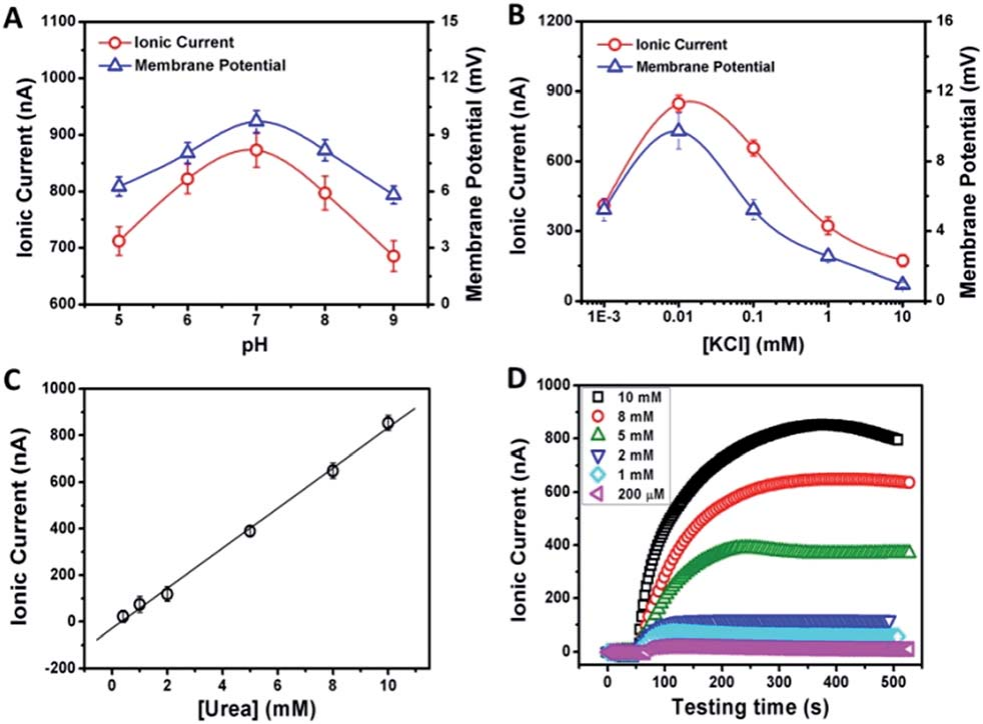
pH (A) and concentration-dependence (B) of the ionic current and membrane potential generated from the urea-driven nanofluidic system. The concentrations of urease and urea were 0.5 mg mL^–1^ and 10 mM, respectively. (C) The dependence of ionic current on urea concentration. (D) Time dependency of ionic currents with respect to the urea concentration (the concentration of urease was 0.5 mg mL^–1^).

As another crucial parameter, the influence of electrolyte concentration was also investigated. The enzymatic catalysis gradually increases with decreasing KCl concentrations from 10 to 0.01 mM (Fig. 3B). This may arise from the fact that higher ion concentration will generate a strong shielding effect on the surface charge of 2D nanochannels, further leading to the weakening of ion-selective transport.^16^ While, as a comparison, too low ionic concentration is also not beneficial for power generation due to the increasing resistance in low-concentration electrolyte. Thus, the optimum electrolyte pH and concentration were chosen to be 7.0 and 0.01 mM, respectively.

The time dependency of ionic current with respect to the urea concentration is shown in Fig. 3C. The concentrations of urease are identical in these measurements (0.5 mg mL^–1^). After adding urea, the ionic current gradually reaches the maximum value within 8 min. The amplitude of the resulting ionic current increases with the urea concentration from 0.2 mM to 10 mM at a constant rate of 82 nA mM^–1^ (Fig. 3C). However, for high-concentration urea above 10 mM, a negative impact to the generated ionic current is observed (Fig. 3D). More urea could not generate larger ionic current. This may be due to that too high a concentration of ammonium ions would increase the interaction between ammonium ions and carboxylate ions, which could result in negative impacts on cation diffusion and power generation.

To examine the possibility of the GPM for practical biowaste conversion, urine from human metabolism was added into the reaction chamber. Fig. 4A and B demonstrate that with the assistance of catalytic reaction, urine can also trigger significant power generation *via* harvesting of both ionic current and generation of membrane potential. The values of current, potential and electric power density are 600 nA, 7 mV and 1.3 mW m^–2^, respectively. The above results reveal practical application for energy conversion using human biowaste. At the present stage, the output voltage is generally less than 10 mV. There is still a long way to go for this technology to be available for most real-world applications. From our point of view, there are two possible strategies to improve the performance of the 2D membrane. First, as key influencing factors, the channel structure and charge density inside the 2D nanochannels could be further tuned through the optimization of film preparation steps. Second, enzymatic catalytic reaction could also be optimized to increase the charge selectivity of the membrane. Further efforts are needed toward a better performance of energy conversion through 2D nanofluidic networks.

**Fig. 4:**
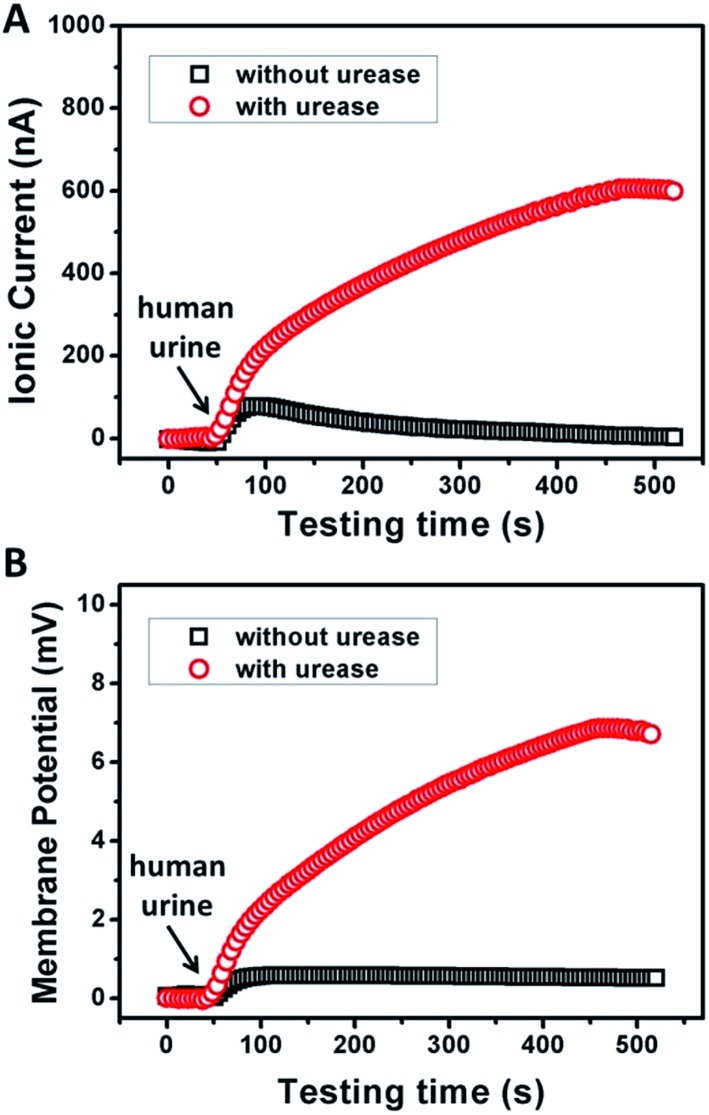
Response of the ionic current (A) and membrane potential (B) of the GPM using human urine triggered enzymatic catalysis. Electrolyte: 0.01 mM KCl, pH 7. The concentration of urease was 0.5 mg mL^–1^.

In conclusion, we demonstrate an energy harvesting device powered by enzymatic biowaste reaction through polyelectrolyte functionalized 2D graphene nanofluidic channels. PAA-functionalized graphene forms a layered structure, and constructs interconnected 2D nanofluidic networks inside the membrane. Negatively charged GPM governs the surface-charge-governed ion transport, preferentially permeating counter-ions while excluding co-ions. Under the force of the enzymatic biowaste reaction, energy conversion is observed in the generation of ionic current and electric potential across the membrane. Our results suggest that the as-proposed 2D graphene–PAA nanosystem shows a sensitive and rapid response to human urine. We believe that the strategy is generally applicable to other types of nanofluidic devices for energy harvesting.

## Supplementary Material

Supplementary information
