# Supplementary material for: Energy harvesting from enzymatic biowaste reaction through polyelectrolyte functionalized 2D nanofluidic channels
Source: Chem Sci. 2016 Feb 16;7(6):3645–8. doi: 10.1039/c5sc04634c (PMC6008704; doi:10.1039/c5sc04634c)
Supplement: Supplementary file 1 [file SC-007-C5SC04634C-s001.pdf]

Electronic Supplementary Material (ESI) for Chemical Science.
This journal is © The Royal Society of Chemistry 2016

Supporting Information

Energy Harvesting From Enzymatic Biowaste Reaction through Polyelectrolyte Functionalized 2D Nanofluidic Channels

Lei Lin, Ling Zhang, Lida Wang, Jinghong Li*

Department of Chemistry, Beijing Key Laboratory for Analytical Methods and Instrumentation, Tsinghua University, Beijing 100084, China

1.Experimental Section

Reagents.

Graphite (99.9 %, 325 mesh) was purchased from Alfa Aesar. Poly(vinylidene fluoride) filter membrane (50 mm in diameter, 0.22 μm pore size) was obtained from Beijing BeiHua Sunrise Membrane Separation Technology Co., Ltd. Polyacrylic acid (PAA, MW: 3000) was gotten from Aladdin Industrial Co.. Urease and urea were Beijing DingGuo Biotech. Co.. Other regents of analytical grade were obtained from Beijing Chemical Company (China). Human urine was obtained from human volunteer. All experiments were were performed in compliance with the relevant laws and institutional guidelines, and were approved by the Institutional Animal Care and Use Committee of Tsinghua University. Informed consent was obtained from the volunteer used in this study.

Synthesis of graphene oxide.

Graphene oxide (GO) was synthesized according to previous reports[1,2]. Generally,

the pre-oxidized graphite was mixed with 12 mL concentrated H_2SO_4 at 0 °C. Then 1.5 g $KMnO_4$ was added slowly under stirring in ice-bath. After stirring at 35 °C for 4 h, 100 mL deionized water was added to dilute the mixture. 2 mL 30% H_2O_2 was then added drop by drop. Next, synthesized GO was filtered and washed with 0.1 M HCl (aq) and deionized water for at least 5 times. The obtained solid was redispersed into deionized water followed by dialysis for 7 days. The resulting solution was stored at 4 °C for further use with the concentration about 2 mg/mL.

Preparation of graphene-PAA composite membranes (GPM).

10 mg GO and 0.2 g PAA were dispersed in 95 mL deionied water by sonication for 0.5 h[3]. Then, 50 μL 80% hydrazine was added and the mixture was kept stirring at 80 °C for 20 h. The resulting mixture was sonicated for 0.5 h and then filtered through a Poly(vinylidene fluoride) filter membrane by vacuum filtration. Finally, the graphene-PAA composite membrane (GPM) was peeled off from the filtration film for further use.

Electrical measurements.

The GPM was mounted between two chambers of the testing cell which were both filled with test buffer (0.01 mM KCl, pH 7.0). Transmembrane potential across the film and streaming current was measured with a Keithley 2636A picoammeter (Keithley Instruments, Inc., Cleveland, OH) through voltmeter mode (Bias I = 0 A) and ammeter mode (Bias V = 0 V), respectively (Scheme S1 in the Supporting Information). Agar-saturated KCl salt bridges were used to connect two pairs of Ag/AgCl electrodes. All the ion current signals were recorded and collected via ACS Basic 1.2 Software (Keithley Instruments, Inc., Cleveland, OH). The average current values at various voltages were obtained through three repeated tests.

2. Schematic image of Electrical Measurement.

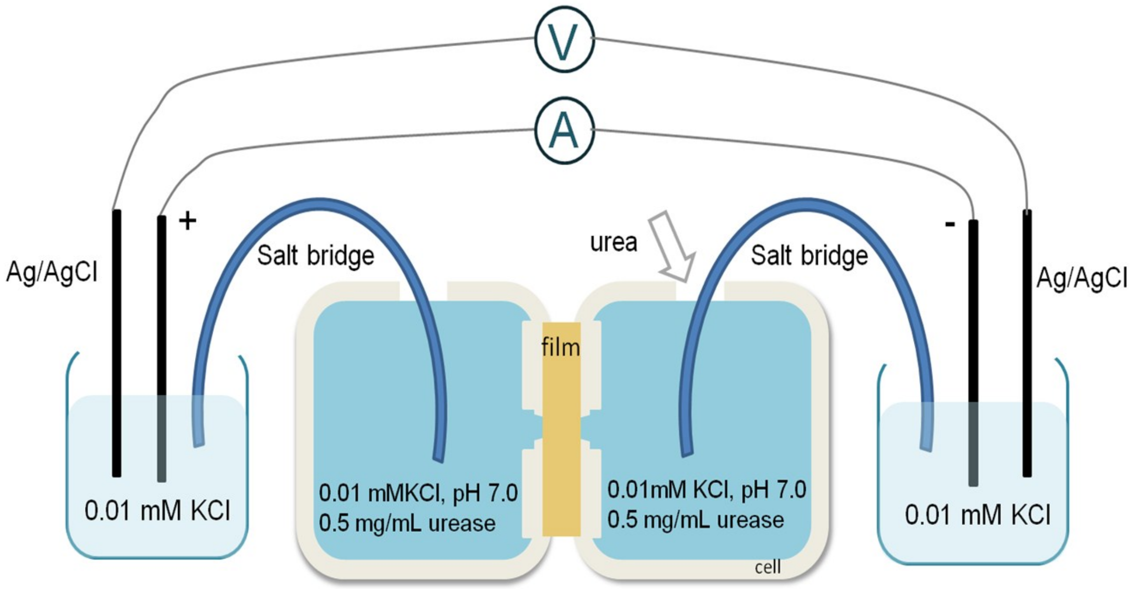

Scheme S1. Schematic image of graphene-PAA composite membranes (GPM) and the cell used for electrical measurement.

3. Characteristics of PAA-functionalized graphene composite membrane.

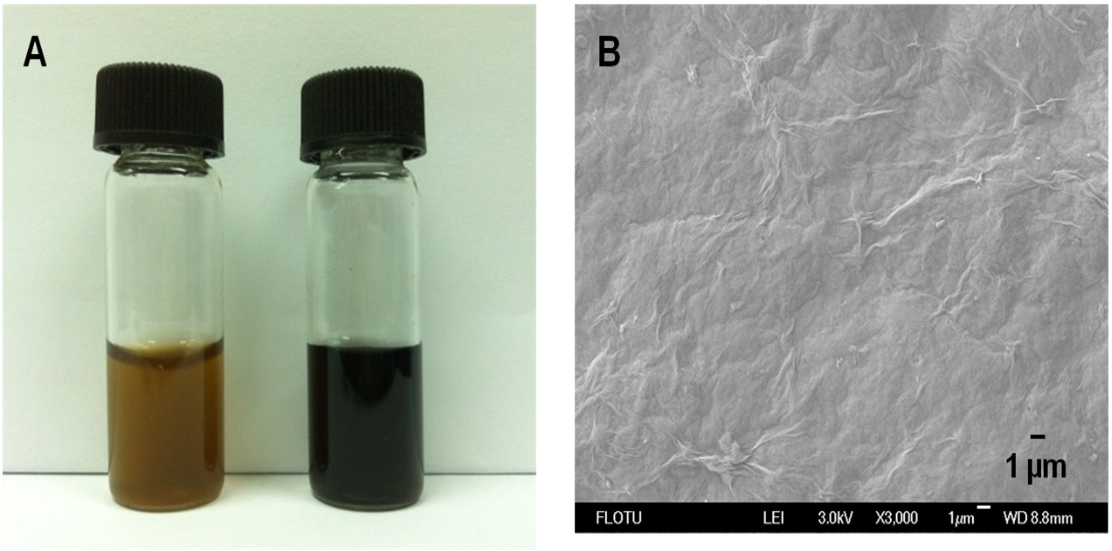

Figure S1. (A) The photographs of GO (left) and graphene-PAA (right) dispersion. (B) SEM image of the surface of a freeze dried GPM.

4. ATR-FTIR spectra characteristics.

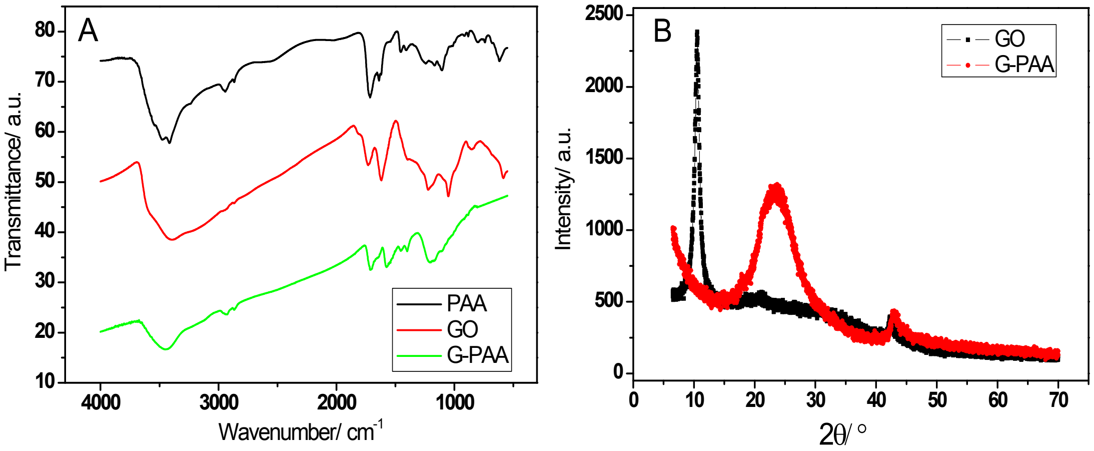

Figure S2. (A) ATR-FTIR spectra of dried GPM. (B) XRD patterns of GO and G-PAA.

The characteristic peaks of GO and graphene-PAA (G-PAA) displayed in the XRD patterns in Figure S2B appeared at 10.5° and 23.8°, respectively. This result indicates that the as-prepared graphene-PAA sheets were largely separated.

5. Zeta potential characteristics of PAA functionalized process.

Table S1. Zeta potential comparison of GO and PAA-functionalized graphene (G-PAA) dispersions.

	Zeta potential/ mV
GO dispersion	-50.6
G-PAA dispersion	-74.1

6. Theoretical calculation of transmembrane electrical potential difference (E_m) and energy conversion efficiency (η_e).

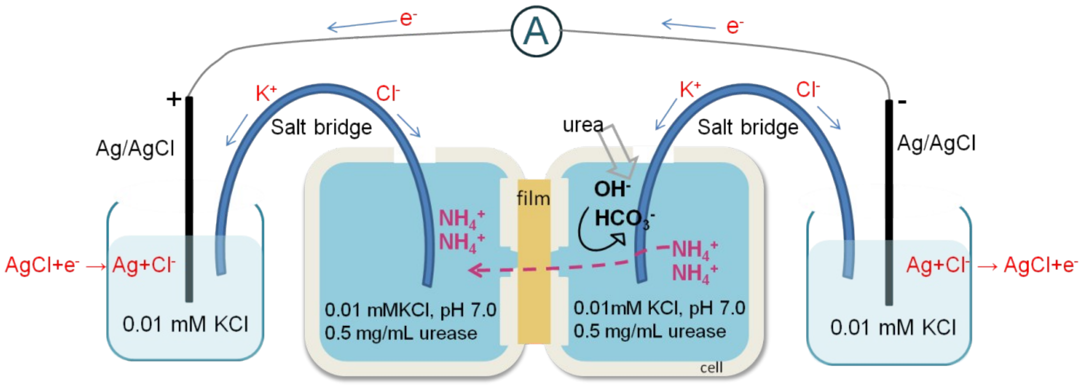

Scheme S2. Schematic representation of transmembrane electrical potential difference (Em). Before adding urea, no ionic concentration gradients occurred and the potentials on both sides of electrodes were same. Thus no electron flowed through the external circuit. After enzymatic catalysis, the *E*m occurred and the net ionic current (Ic) generated by redox reactions on electrodes.

Scheme S2 displayed the principle of the current conversion. When the urea molecules are catalyzed by urease, the yielding NH_4^+ goes across the membrane under the chemical gradients from the reaction chamber to the receive chamber. The resulting charge imbalance across the membrane (measured as an transmembrane electrical potential difference, Em) could be calculated according to the following Equation (1) :

$$E_m = |E^C - E^N| = \left|\left(E^{C\theta} + \frac{RT}{zF} ln\frac{\alpha_{ox}^{C}}{\alpha_{re}^{C}}\right) - \left(E^{N\theta} + \frac{RT}{zF} ln\frac{\alpha_{ox}^{N}}{\alpha_{re}^{N}}\right)\right| \qquad (1)$$

Where E^C and E^N are electrode potentials at each side of enzymatic catalysis and non-catalysis respectively, F is the Faraday constant, T is the absolute temperature (K), R is the gas constant, and α^C and α^N are activities of the products that exist in the catalysis and non-catalysis side of the cell. To Ag/AgCl electrode, $E^{C\theta}$ and $E^{N\theta}$ were equal, thus Equation (1) could be substituted with Equation (2) :

$$E_m = \left|\frac{RT}{F} ln\frac{\alpha_{AgCl}^{C}}{\alpha_{Ag}^{C}\alpha_{Cl^-}^{C}} - \frac{RT}{F} ln\frac{\alpha_{AgCl}^{N}}{\alpha_{Ag}^{N}\alpha_{Cl^-}^{N}}\right| \qquad (2)$$

In order to simplify the calculation, α_{Ag} and α_{AgCl} could be removed and α_{Cl^-} were

equal to the Cl^- concentration, Equation (3) was finally obtained:

$$E_m = \left|\frac{RT}{F} ln \frac{[Cl^-]^N}{[Cl^-]^C}\right| = \left|0.059 lg \frac{[Cl^-]^N}{[Cl^-]^C}\right| \qquad (3)$$

In our experimental setup, the concentrations of electrolyte and urea were 0.01 mM and 10 mM, respectively. Assuming that urea would be fully catalyzed into NH_4^+ by urease, E_m could be estimated according to the Equation (3) and the maximum voltage was calculated to be 177 mV (absolute value). In this case, the efficiency can be further obtained based on the following equation:

$$\eta_e = \frac{P_{mea}}{P_{cal}} = \frac{I \cdot V_{mea}}{I \cdot V_{cal}} = \frac{V_{mea}^2/R}{V_{cal}^2/R} = \frac{V_{mea}^2}{V_{cal}^2} \qquad (4)$$

where P_{cal} and P_{mea} are the calculated and measured power, corresponding to the calculated and measured voltages. Thus we can calculate the efficiency η_e and the value was around 0.3%.

7. Long time testing for the response of the ionic current under the urea triggered enzymatic catalysis.

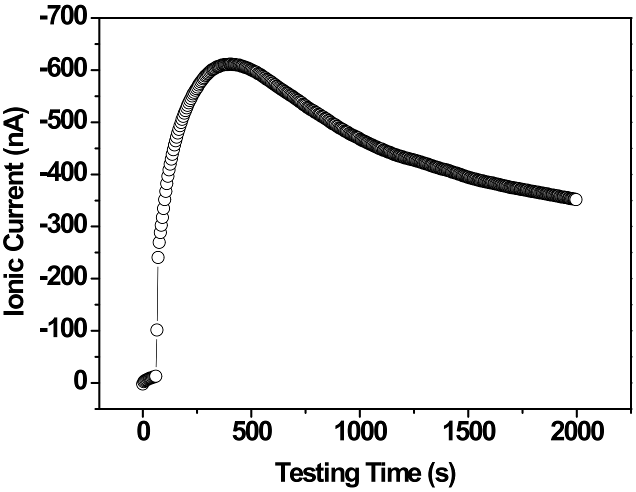

Figure S3. Long time response of the ionic current of GPM under the urea triggered enzymatic catalysis. Electrolyte: 0.01 mM KCl, pH 7.

In order to investigate the long-time response of ionic currents, a period of 2000 s testing time were applied for the experiment. It is clearly observed that ionic current gradually decreased with the testing time increasing. It might be attributed to the

decrease of concentration gradient between the chambers after the complete enzymatic catalysis and urea degradation.

References

(1) Hummers, W. S.; Offeman, R. E. *J. Am. Chem. Soc.* **1958**, *80*, 1339.

(2) Tang, L. H.; Feng, H. B.; Cheng, J. S.; Li, J. H. *Chem. Commun.* **2010**, *46*, 5882.

(3) Lee, J. Y.; In, I. *Chem. Lett.* **2012**, *41*, 127.
